# Auditing the Early Management of Suspected Scaphoid Fractures in Comparison With the National Agreed Standard: A Retrospective Study

**DOI:** 10.7759/cureus.98228

**Published:** 2025-12-01

**Authors:** Thomas M Downs, Mahir Yousuff, Arun Paul, Catherine Oliver, Abdullah Ashfaq, Ian Stewart, Arham Khokhar, Amit Bidwai

**Affiliations:** 1 Trauma and Orthopaedics, Sherwood Forest Hospitals NHS Foundation Trust, Sutton-in-Ashfield, GBR

**Keywords:** diagnosis of scaphoid fracture, management scaphoid fracture, scaphoid fracture, scaphoid fracture treatment, suspected scaphoid fracture

## Abstract

Introduction

Scaphoid fractures are the most common carpal bone injury and a frequent reason for wrist immobilisation following trauma. However, many patients with clinical suspicion of scaphoid fracture are immobilised despite negative radiographs, and ultimately have alternative diagnoses. This study aimed to evaluate early management, imaging, and outcomes for patients presenting with suspected scaphoid fractures at a District General Hospital (DGH) in the United Kingdom (UK), comparing practice with current British Society for Surgery of the Hand (BSSH) standards.

Methods

A retrospective audit was conducted of all adults presenting between January 2023 and December 2024 with suspected scaphoid fracture. Primary audit outcomes included compliance with seven-day referral to the fracture clinic, and whether a four-view scaphoid radiograph series was performed. Secondary and additional cohort data were collected on demographics, clinical features, and outcomes.

Results

A total of 248 patients were included (mean age 47.5 years; 56.5% female). Most injuries resulted from a fall on an outstretched hand (n = 208, or 83.9%). Review within seven days occurred in 68.5% (n = 170) of cases, and 95.2% (n = 236) underwent a dedicated four-view scaphoid radiograph series. A scaphoid fracture was confirmed in 44 patients (17.7%), while 82.3% had alternative diagnoses. The false-negative rate of initial radiographs was 22.7% (10/44; 95% CI 11.5-37.8%), and the false-positive rate was 5.2% (13/248; 95% CI 2.8-8.8%). Advanced imaging was obtained in 29.0% of patients, including computed tomography (CT) and magnetic resonance imaging (MRI), with median time at 36.5 and 30 days, respectively. The median time to exclusion of a scaphoid fracture by a consultant in a specialist orthopaedic clinic was 18.5 days (mean 27.5 days). Among confirmed fractures, 88.6% were treated conservatively, and 95.5% achieved union. There were no cases of avascular necrosis.

Conclusion

Although compliance with early review and imaging standards was high, most patients were immobilised without a true fracture, reflecting persistent overtreatment. Earlier MRI access, improved documentation, and clearer immobilisation protocols may reduce unnecessary treatment, enhance diagnostic efficiency, and strengthen alignment with national standards for suspected scaphoid fractures.

## Introduction

Following wrist trauma, scaphoid fractures represent 51%-80% of all carpal bone fractures and are a common cause of emergency department (ED) attendances [1,2]. Fractures typically occur after a fall onto an outstretched hand (FOOSH) and are of particular clinical pertinence due to the scaphoid’s precarious retrograde blood supply. Therefore, the scaphoid has an increased risk of avascular necrosis, delayed union, and non-union, especially in proximal pole fractures [3].

Diagnosing scaphoid fractures early remains challenging, with up to 21.8% of fractures occult on initial radiographs, leading many EDs to treat them as ‘fracture until proven otherwise’ [4]. Consequently, many patients are immobilised unnecessarily, despite the risk-averse intention of this approach. Research has found that only 6% of patients with clinically suspected fractures had a confirmed scaphoid fracture, yet most were immobilised for approximately 30 days [5]. Previous research has shown that only 71% of centres in the United Kingdom (UK) have established guidelines for the management of suspected scaphoid fractures, highlighting considerable variability in clinical practice [6,7]. The National Institute for Health and Care Excellence (NICE) [8] suggests magnetic resonance imaging (MRI) as first line for suspected scaphoid fractures when there is diagnostic doubt; however, early access remains limited [9]. 

Recent randomised controlled trial (RCT) evidence from the ‘Clinically SUspected ScaPhoid fracturE: treatment with supportive bandage or casT’ (SUSPECT) study has questioned whether routine casting is indicated in patients with non-concerning initial radiographs. The study demonstrated non-inferior outcomes with supportive bandaging and early review [10]. However, many institutions continue to follow traditional immobilisation pathways pending specialist orthopaedic fracture clinic review and further imaging.

This retrospective audit aimed to describe local practice in the management of patients presenting with suspected scaphoid fracture to a National Health Service (NHS) District General Hospital (DGH). It audited compliance with certain British Society for Surgery of the Hand (BSSH) [11] standards, whilst further observational analyses described imaging strategy, immobilisation, and clinical outcomes.

## Materials and methods

This was a single-centre, retrospective audit conducted at Kings Mill Hospital, a 650-bed DGH in the UK. The study aimed to assess the number of potential scaphoid fractures that were subsequently confirmed as true scaphoid fractures. Inclusion criteria were adults aged 18 and over who presented to the ED with a new, potential scaphoid fracture during the two-year study period, from January 1, 2023, to December 31, 2024. Exclusion criteria included any presentation where the patient was already under follow-up for a previously diagnosed scaphoid fracture, paediatric patients under 18 years of age, and patients in whom there was no clinical coding of a scaphoid fracture (for example, those with hand or distal radius injuries). The initial patient list was extracted via SystmOne (The Phoenix Partnership, Leeds, UK), an Electronic Health Record (EHR) software used across NHS trusts. Further demographic and clinical information was obtained via the hospital’s Picture Archiving and Communication System (PACS), alongside outpatient clinic letters. This audit was approved by the clinical governance team at Sherwood Forest Hospitals NHS Foundation Trust, Sutton-in-Ashfield, UK, under code T&O/CA/2025-26/05. In accordance with UK Health Research Authority guidance, an Institutional Review Board (IRB) or Research Ethics Committee (REC) was not required for this local clinical audit. 

Typical patient pathway

Initial Management

The typical patient pathway for a potential scaphoid fracture is best described by the BSSH and is broadly summarised here. In our study, we have referred to the BSSH over the NICE standards, given its extended scope. Patients usually present to the ED and are assessed by a clinician after a forced wrist hyperextension injury, such as a fall on an outstretched hand. Typical clinical findings include tenderness in the anatomical snuffbox, pain on axial loading of the thumb, or tenderness over the scaphoid tubercle. They will undergo a four-view scaphoid radiograph series (posterior-anterior view, posterior-anterior view with ulnar deviation, lateral, and oblique). If a fracture is visible, they will have a below-elbow backslab. If a fracture is not visible, it will be immobilised in a wrist splint. Both groups of patients will be followed up in a specialist fracture clinic, usually within seven days, for further clinical assessment by an orthopaedic surgeon, and additional imaging as required. 

Specialist Management

If a scaphoid fracture is ruled out through a combination of clinical examination and possibly further imaging, including serial radiographs, computed tomography (CT), or MRI, the patient can remove any immobilising splint and be discharged as appropriate. This may include referral to hand therapy or patient-initiated follow-up (PIFU). Patients with confirmed scaphoid fractures will either be managed with acute surgical fixation or non-operatively in a below-elbow cast. They will be followed up regularly until radiographic union and clinical improvement are achieved.

Data analysis

Primary Audit Outcomes

We audited local practice against the BSSH Scaphoid Standards. The primary audit outcomes were compliance with review in a specialist fracture clinic within seven days of presentation, and compliance with obtaining a dedicated four-view scaphoid radiograph series at initial assessment.

The presence of a documented definitive management plan within 14 days was not audited, because this standard applies specifically to patients with a genuine suspicion of scaphoid fracture, whereas in our study, many patients were referred to the clinic as a means of a safety net for their wrist injury. Hence, assessing compliance with a 14-day management plan in an inexact patient group would have led to error. Furthermore, interpreting what was recorded in the clinical notes as 'definitive' management can be inconsistent. 

Secondary Outcomes and Additional Descriptive Data

Secondary outcomes included the false-positive and false-negative rates of initial radiographs (see 'Definitions' below), the type of initial immobilisation, the time to specialist exclusion of scaphoid fracture, the time to additional imaging of CT and MRI, and the proportion of confirmed fractures that achieved union.

Further data were collected among two groups of patients: those with suspected scaphoid fracture and those with confirmed scaphoid fracture. From suspected scaphoid fracture patients, variables were collected on demographics, injury details, clinical examination findings at initial fracture clinic, need for further imaging, diagnosis, and referral to hand therapy. For confirmed scaphoid fractures, variables were collected on the type of initial immobilisation, location, conservative or surgical management, and outcomes.

Definitions: A 'false positive' radiograph was defined as an initial four-view scaphoid radiograph series interpreted as showing a scaphoid fracture, which was later excluded by advanced imaging or specialist assessment. This is divided by the denominator, which is the full cohort of patients presenting with suspected scaphoid fracture (n = 248), to give the 'false-positive rate'.

A 'false negative' radiograph was defined as an initial four-view scaphoid radiograph series showing no fracture in a patient who was subsequently confirmed to have a scaphoid fracture. This is divided by the denominator, which is all patients with a confirmed scaphoid fracture (n = 44), to give the 'false-negative rate'.

Statistical analysis

Data analysis was performed using Microsoft Excel (Microsoft Corporation, Redmond, WA, USA) and GraphPad Prism (version 9; GraphPad Software, San Diego, CA, USA). Missing data were recorded as ‘N/A’ and were due to absent documentation in the clinical record. As this was a clinical audit, analyses were descriptive; exact binomial (Clopper-Pearson) 95% confidence intervals (CI) were calculated for key proportions, and no formal hypothesis testing was undertaken. Percentages are given to one decimal place, and where relevant, mean, median, and interquartile range (IQR) are used. Tables were used to summarise demographic and clinical characteristics, and outcomes. Graphical representations of select categorical variables were performed using bar charts and pie charts. 

## Results

A total of 248 patients met the inclusion criteria over the two-year study period. Table 1 summarises primary and secondary audit outcomes. Review in a specialist fracture clinic within seven days was achieved in 68.5%, and 95.2% underwent a dedicated four-view scaphoid radiograph series at initial presentation.

**Table 1:** Primary and secondary outcomes Primary audit outcomes compared with BSSH targets, and secondary outcomes describing diagnostic accuracy, immobilisation, imaging timing, and fracture union. *False-negative rate and union rate are calculated from the confirmed scaphoid fracture group (n = 44). All other variables are divided by the entire cohort (n = 248). BSSH, British Society for Surgery of the Hand; IQR, Interquartile Range; CT, Computed Tomography; MRI, Magnetic Resonance Imaging

Primary Audit Outcomes		
Variable	Achieved n (%)	Target n (%)
Review in clinic in 7 days	170 (68.5)	248 (100)
Initial four-view scaphoid radiograph series	236 (95.2)	248 (100)
Secondary Outcomes		
Variable	Category	n (%) or days
Initial radiograph	False positive rate	13 (5.2)
	False negative rate*	10 (22.7)
Type of initial immobilisation	Splint	185 (74.6)
	Backslab	49 (19.8)
	None	14 (5.6)
Time to specialist exclusion of scaphoid fracture	Mean	27.5 days
	Median (IQR)	18.5 (9-39.5) days
Median time (IQR) to further imaging	CT	36.5 (19.5-68.8) days
	MRI	30 (20-39.8) days
Proportion of confirmed scaphoid fractures that united*		42 (95.5)

Regarding the diagnostic utility of initial radiographs, an initial positive radiograph for scaphoid fracture resulted in no fracture for 13 patients, giving a false-positive rate of 5.2% (95% CI 2.8-8.8%). An initial negative radiograph resulted in a scaphoid fracture in 10 patients, giving a false-negative rate of 22.7% (95% CI 11.5-37.8%). The type of initial immobilisation was as follows: 185 patients (74.6%) splint, 49 patients (19.8%) backslab, and 14 patients (5.6%) with none. The time to definitive exclusion of a scaphoid fracture by a consultant orthopaedic surgeon was a median of 18.5 days (IQR 9-39.5 days) and a mean of 27.5 days. The median times to further imaging were 36.5 days (19.5-68.8) for CT scan and 30 days (20-39.8) for MRI. Of the 44 confirmed scaphoid fractures, 42 patients (95.5%) achieved union. 

Initial demographic and clinical features are shown in Table 2. The mean age was 47.5 years, with 108 males (43.5%) and 140 females (56.5%). Injuries involved the right wrist in 115 cases (46.4%) and the left in 133 (53.6%). The dominant hand was affected in 45 patients (18.1%), the non-dominant in 37 (14.9%), and was unrecorded in 166 (66.9%). Most injuries resulted from a fall on an outstretched hand (n = 208; 83.9%), followed by hyperextension (n = 12; 4.8%) and other mechanisms (n = 28; 11.3%). Review in the fracture clinic within seven days occurred in 170 patients (68.5%), consistent with national standards.

**Table 2:** Initial demographic and clinical features This table represents baseline characteristics and clinical presentation of all patients (n = 248) presenting with suspected scaphoid injury over a two-year period. Values are expressed as numbers (percentages) unless otherwise indicated. FOOSH, Fall Onto an Outstretched Hand; N/A, Not Available

Variable	Category	n (%)
All patients		248
Age	Mean	47.5 years
Gender	Male	108 (43.5)
	Female	140 (56.5)
Side of injury	Right	115 (46.4)
	Left	133 (53.6)
Dominant hand involved	Yes	45 (18.1)
	No	37 (14.9)
	N/A	166 (66.9)
Mechanism of injury	FOOSH	208 (83.9)
	Hyperextension	12 (4.8)
	Other	28 (11.3)
Initial fracture clinic in 7 days	Yes	170 (68.5)
Initial radiographs	Four-view scaphoid radiograph series	236 (95.2)
	Fracture present	47 (19.0)
	No fracture seen	201 (81.0)
Initial immobilisation	Splint	185 (74.6)
	Backslab	49 (19.8)
	None	14 (5.6)
Snuffbox tenderness	Yes	162 (65.3)
	No	53 (21.4)
	N/A	33 (13.3)
Scaphoid tubercle tenderness	Yes	93 (37.5)
	No	66 (26.6)
	N/A	89 (35.9)
Pain axial thumb loading	Yes	45 (18.1)
	No	58 (23.4)
	N/A	145 (58.5)

**Figure 1:**
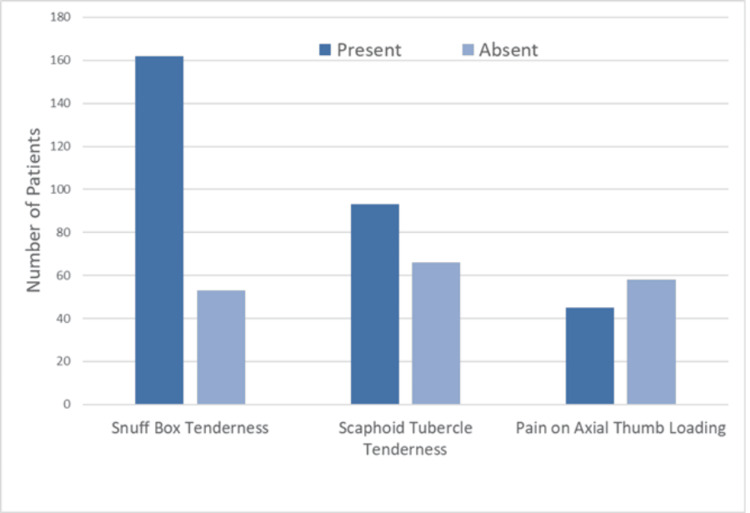
Clinical examination findings at initial presentation Proportion of patients with documented anatomical snuffbox tenderness, scaphoid tubercle tenderness, and pain on axial thumb loading. Excludes cases with no recorded data.

**Figure 2:**
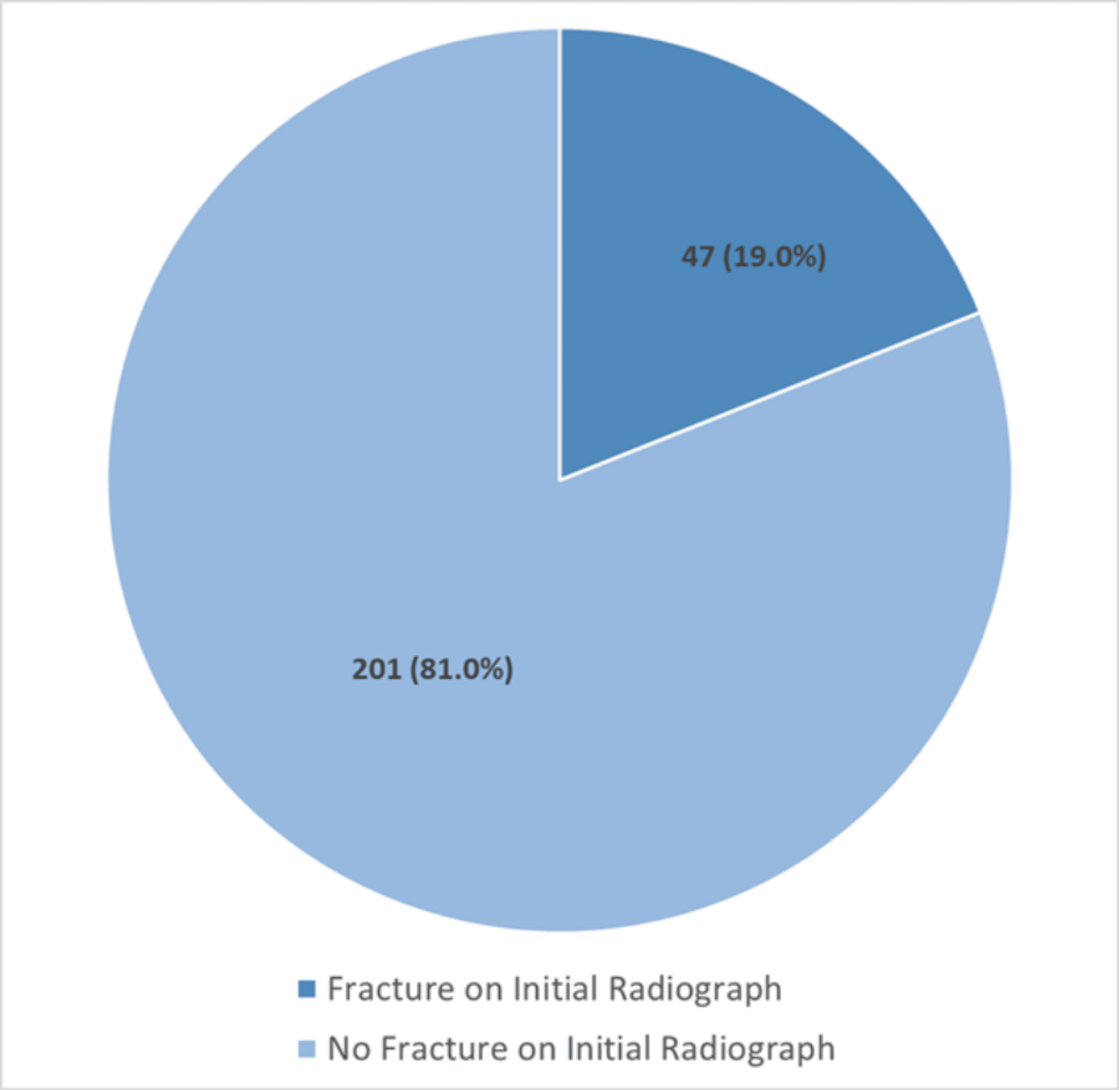
Initial radiographic assessment and detection rate Number of patients undergoing a dedicated four-view scaphoid radiograph series and proportion with a visible fracture on initial plain radiographs.

Clinical signs were variably documented (Figure 1): snuffbox tenderness was positive in 162 patients (65.3%), scaphoid tubercle tenderness in 93 patients (37.5%), and pain on axial thumb loading in 45 patients (18.1%). Dedicated four-view scaphoid radiographs were performed in 236 patients (95.2%), with a visible fracture in 47 (19.0%) and no visible fracture in 201 (81.0%) (Figure 2).

**Figure 3:**
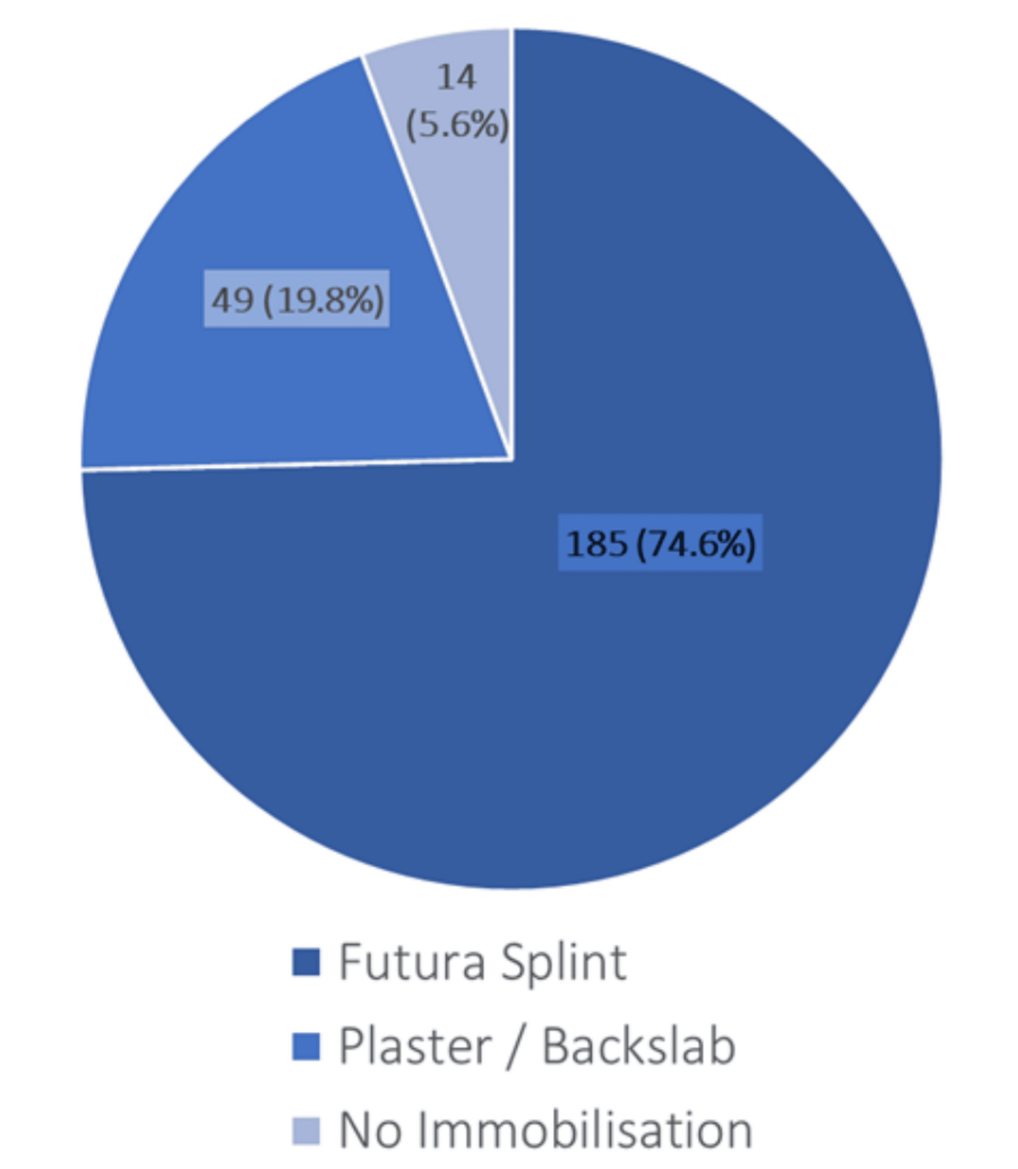
Initial immobilisation Distribution of patients managed with wrist splint, below-elbow backslab, or no immobilisation after initial presentation.

Following assessment, 185 patients (74.6%) were immobilised in a wrist splint, 49 (19.8%) in a below-elbow backslab, and 14 (5.6%) had no immobilisation recorded (Figure 3).

**Figure 4:**
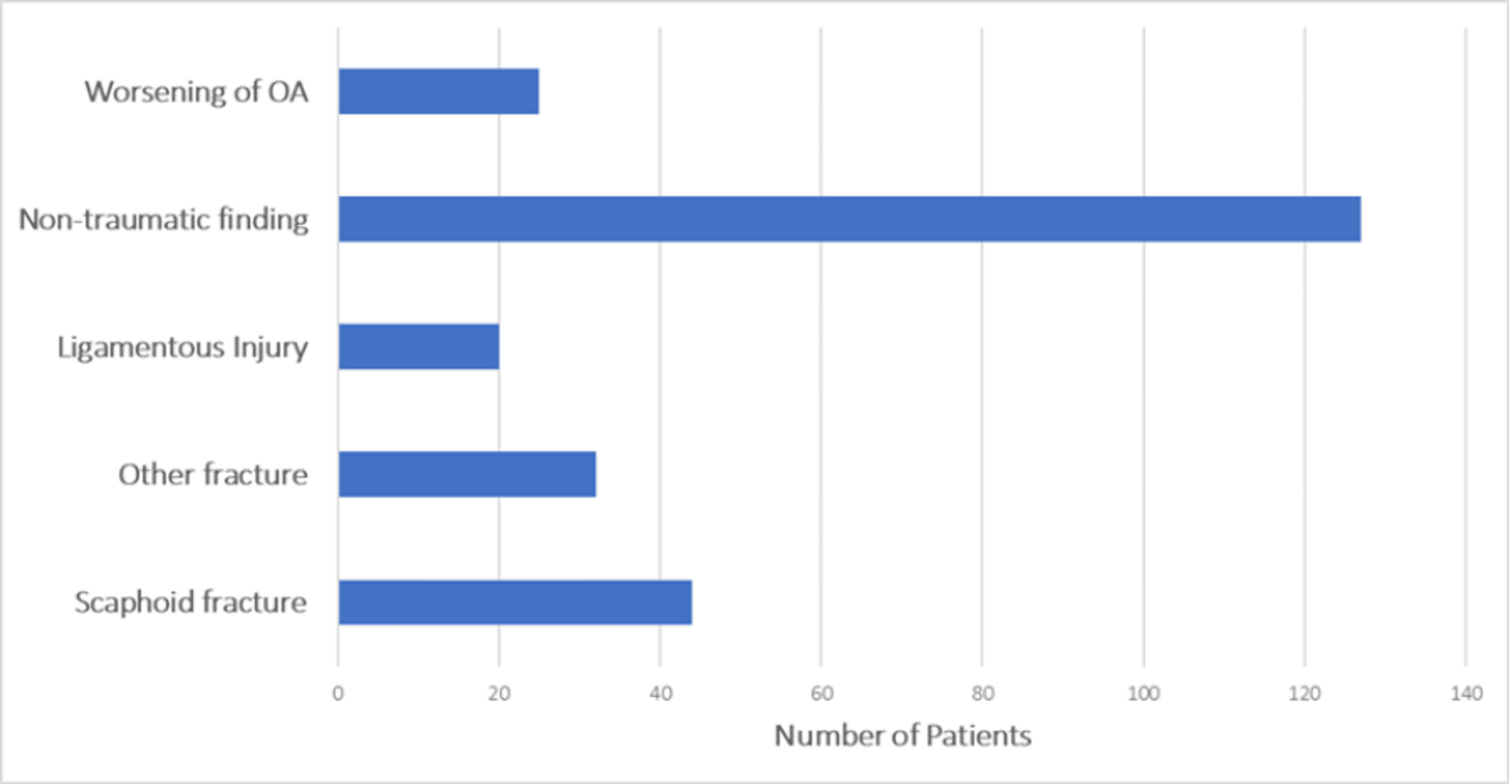
Diagnostic findings Breakdown of definitive diagnoses, including confirmed scaphoid fracture, other fractures, ligamentous injuries, non-traumatic findings, and osteoarthritis (OA).

Diagnostic findings are shown in Figure 4 and Table 3. A scaphoid fracture was ultimately confirmed in 44 patients (17.7%), while 32 (12.9%) had other fractures, 20 (8.1%) ligamentous injuries, 25 (10.1%) worsening osteoarthritis, and 127 (51.2%) non-traumatic or soft-tissue findings. Other fractures mostly included non-scaphoid carpal fractures and distal radius fractures. Ligamentous injuries were described as such by an orthopaedic surgeon and include scapholunate ligament injuries, triangular fibrocartilage complex tears, and isolated tendon injuries, such as the extensor carpi ulnaris. Non-traumatic findings include non-identifiable pathologies, such as wrist sprain.

**Table 3:** Cohort and scaphoid fracture outcomes This table describes diagnostic, management and outcome data for the study cohort and scaphoid fracture cohort. Union and non-union outcomes are based on radiological confirmation at follow-up. OA, Osteoarthritis; IQR, Interquartile Range; CT, Computed Tomography; MRI, Magnetic Resonance Imaging

Variable	Category	n (%)
All patients		248
Further imaging needed	CT	29 (11.7)
	MRI	43 (17.3)
Median time (IQR) to further imaging	CT	36.5 (19.5-68.8) days
	MRI	30 (20-39.8) days
Initial radiograph positive but non-scaphoid fracture	False positive rate	13 (5.2)
Diagnosis	Scaphoid fracture	44 (17.7)
	Other fracture	32 (12.9)
	Ligamentous injury	20 (8.1)
	Non-traumatic finding	127 (51.2)
	Worsening of OA	25 (10.1)
Average time to specialist exclusion of scaphoid fracture	Mean	27.5 days
	Median (IQR)	18.5 (9-39.5) days
Referral to hand therapy	Yes	79 (31.9)
Scaphoid fracture outcomes		n = 44
Diagnosed after negative initial radiograph	False negative rate	10 (22.7)
Type of initial immobilisation	Splint	17 (38.6)
	Plaster	27 (61.4)
Location	Waist	35 (79.5)
	Proximal pole	0 (0.0)
Management	Conservative	39 (88.6)
	Surgical	5 (11.4)
Outcomes	Union	42 (95.5)
	Non-union	2 (4.5)
	Avascular necrosis	0 (0.0)

Advanced imaging was obtained in 72 patients (29.0%), including 29 CT and 43 MRI scans, with the median time for both beyond 14 days.

Among confirmed scaphoid fractures, 17 patients (38.6%) were initially immobilised in a splint, and 27 (61.4%) in plaster. Thirty-five (79.5%) were scaphoid waist fractures. Thirty-nine (88.6%) were managed conservatively, and five (11.4%) underwent surgical fixation. Union occurred in 42 cases (95.5%), with two instances (4.5%) of non-union (at six months), and no cases of avascular necrosis. Seventy-nine patients (31.9%) of the initial 248 patients were referred for hand therapy during recovery.

## Discussion

This retrospective audit evaluated local compliance with two key BSSH Scaphoid standards: timely review in a specialist fracture clinic within seven days, and completion of a dedicated four-view scaphoid radiograph series at initial assessment, with compliance high for both standards. This study also describes secondary and additional cohort data, such as radiographic accuracy, immobilisation patterns, imaging time, and outcomes in confirmed fractures. Nearly all patients underwent immobilisation but had alternative diagnoses, whilst those with true scaphoid fractures were stable, with high rates of union. Although limited by a single-centre, retrospective design, with variability in documentation, this study provides a valuable snapshot of current practice.

Demographically, our results show that most patients presenting with suspected scaphoid fractures are middle-aged adults following a FOOSH, reflecting the general wrist injury population [12], in contrast to true scaphoid fractures, which occur commonly in younger men [13,14]. As expected, most patients (68.5%) were reviewed in the fracture clinic within seven days, with nearly all (95.2%) undergoing a dedicated four-view scaphoid radiograph series, demonstrating good compliance with recommended early review and imaging standards (BSSH). Despite this, initial radiographs predicted a scaphoid fracture when there was not one present - a false-positive rate - in 13 (5.2%) patients from the original cohort of 248. This is in line with Jain et al., who reported an overdiagnosis of 18 out of 114 patients [15]. Conversely, the false-negative rate was 22.7%, which is 10 patients out of 44 who had a true scaphoid fracture but a negative initial radiograph. This is equivalent to the 21.8% of radiologically occult fractures reported in a meta-analysis of 42 studies by Bäcker et al. [4], which also included four-view radiographs on the day of injury. Reasons for misdiagnosis can include degenerative changes, irregular tubercles, or, possibly in fresh injuries, not enough time elapsed for a trabecular injury to cause a cortical or radiolucent line [16]. In our study, 25 (10.1%) patients had worsening degenerative changes and a final diagnosis of osteoarthritis, similar to the literature [17]. Only a minority of patients proceeded to CT or MRI, and most of these scans occurred beyond the 14-day timeframe in which the BSSH recommends that a definitive management plan should be made. RCT evidence suggests MRI directly from the ED produces cost savings at six months, with higher patient satisfaction and reduced immobilisation [18]. Dean [6] suggests the reason why centres cannot provide early MRI access is multifactorial, from reduced scanning capacity to poor integration of pathways and services. This audit, therefore, identifies an opportunity to streamline early imaging pathways to likely reduce overtreatment. 

The most frequently documented clinical test at initial presentation was anatomical snuffbox tenderness. Parvizi et al. [19] reported that snuffbox tenderness, scaphoid tubercle tenderness, and pain on axial thumb loading are equally useful for diagnosing scaphoid fractures, although the relative frequency of each was not specified. Huynh et al. [20] estimate that the probability of fracture is 60% when all three are positive after a wrist injury. More recently, a systematic review by Coventry et al. [21], analysing 1,685 wrist injuries, found that supination against resistance was the most accurate clinical predictor of occult scaphoid fracture, with a likelihood ratio (LR) of +45, while the absence of anatomical snuffbox tenderness significantly reduced probability, with an LR of -0.2. A limitation in our study, however, was the incomplete documentation or assessment of these tests. The dominant hand was similarly assessed but lacked complete data. Hill et al., in an audit of 4,873 hand and wrist injuries, found that the dominant hand was more commonly injured, attributed to the fact that people may use their hand to brace during a fall [22]. Early referral to hand therapy is essential to restore wrist motion and function following immobilisation; although only 79 patients (31.9%) were referred in our study, many were given exercises to perform by themselves.

The overall scaphoid fracture rate of 17.7% lies toward the upper end of the literature range of 6%-16% [5,10,23], possibly reflecting local variation. In terms of initial immobilisation, 74.6% were splinted, 19.8% were in plaster, and non-scaphoid fractures awaited a median of 18.5 days to reach a final diagnosis. Shetty et al. found that, in 2011, 84% were immobilised in casts whilst awaiting an average of 30.6 days in a Welsh population [5]. This shows that our current practice adheres favourably to BSSH guidelines, and possibly reflects that the guidelines themselves have evolved to avoid the stiffness and burden of prolonged cast immobilisation. The study by Langhoff and Andersen found similar union rates, provided immobilisation by cast is started within four weeks [24], offering some reassurance to practitioners fearful of a delayed diagnosis, such as in our study, where 17 (38.6%) scaphoid fractures were initially splinted instead of plaster from the ED. Furthermore, occult fractures are more likely to be non-displaced waist or distal pole fractures, which are less likely to result in non-union than proximal pole fractures [24]. Our study was reassuring in that there was a 95.5% union rate and no cases of avascular necrosis.

This study has several limitations inherent to its design. As a single-centre, retrospective audit, it is subject to documentation variability and incomplete data capture, particularly regarding clinical examination findings and hand dominance. The results, therefore, reflect local practice and may not be fully generalisable to other settings. Additionally, not all patients underwent cross-sectional imaging, which could have led to underestimation of occult fracture rates. Despite these constraints, the large sample size and inclusion of the full patient pathway provide a realistic overview of everyday suspected scaphoid fracture management in a typical UK DGH.

Future work could explore the development of an updated 'suspected scaphoid' pathway, with earlier access to MRI for patients with negative radiographs but persistent clinical suspicion, supported by complete and standardised documentation and clearer immobilisation guidance. Specifically, a definitive plan, with the possible inclusion of MRI, within 14 days, could be appropriately audited. Further interventions could include more training for primary clinicians to help distinguish those with wrist injuries from genuinely suspected scaphoid fractures. Multi-centre evaluation may help assess the impact of such changes on diagnostic efficiency, immobilisation duration, and adherence to BSSH standards. Prospective studies would allow for deeper statistical analyses and exploration of associations beyond the descriptive scope of this audit.

## Conclusions

This audit provides an up-to-date overview of early management practices for suspected scaphoid fractures in a UK DGH. While compliance with initial assessment and imaging standards was high, most patients were immobilised without a confirmed fracture, highlighting opportunities to improve diagnostic accuracy and reduce unnecessary treatment. Greater use of early MRI, consistent documentation, and clearer immobilisation protocols may enhance efficiency, optimise patient outcomes, and strengthen adherence to national standards.
